# Quality of family history collection with use of a patient facing family history assessment tool

**DOI:** 10.1186/1471-2296-15-31

**Published:** 2014-02-13

**Authors:** R Ryanne Wu, Tiffany L Himmel, Adam H Buchanan, Karen P Powell, Elizabeth R Hauser, Geoffrey S Ginsburg, Vincent C Henrich, Lori A Orlando

**Affiliations:** 1 Health Services Research and Development, Department of Veteran Affairs Medical Center, 411 W. Chapel Hill St., Ste 600, Durham, NC 27701, USA; 2 Duke Center for Personalized and Precision Medicine, Institute of Genome Science & Policy, Duke University, 2111 Ciemas, 101 Science Dr., DUMC Box 3382, Durham, NC 27708, USA; 3 Duke Department of Internal Medicine, Duke University Health System, Durham, NC, USA; 4 Duke Cancer Institute, Duke University Health System, Seeley Mudd Building, 10 Bryan Searle Drive, DUMC Box 3917, Durham, NC 27710, USA; 5 Center for Biotechnology, Genomics and Health Research, UNC-Greensboro, 3701 Moore Humanities and Research Administration Building, 1111 Spring Garden Street, Greensboro, NC 27412, USA; 6 CSP Epidemiology Center, Department of Veteran Affairs Medical Center, 411 W. Chapel Hill St., Ste 700, Durham, NC 27701, USA; 7 Center for Human Genetics, DUMC, Duke University, Box 3445, Durham, NC 27710, USA

**Keywords:** Family history, Data quality, Patient-centered

## Abstract

**Background:**

Studies have shown that the quality of family health history (FHH) collection in primary care is inadequate to assess disease risk. To use FHH for risk assessment, collected data must have adequate detail. To address this issue, we developed a patient facing FHH assessment tool, MeTree. In this paper we report the content and quality of the FHH collected using MeTree.

**Methods:**

*Design:* A hybrid implementation-effectiveness study. Patients were recruited from 2009 to 2012. *Setting:* Two community primary care clinics in Greensboro, NC. *Participants:* All non-adopted adult English speaking patients with upcoming appointments were invited to participate. *Intervention:* Education about and collection of FHH with entry into MeTree. *Measures:* We report the proportion of pedigrees that were high-quality. High-quality pedigrees are defined as having all the following criteria: (1) three generations of relatives, (2) relatives’ lineage, (3) relatives’ gender, (4) an up-to-date FHH, (5) pertinent negatives noted, (6) age of disease onset in affected relatives, and for deceased relatives, (7) the age and (8) cause of death (*Prim Care***31:**479–495, 2004.).

**Results:**

Enrollment: 1,184. Participant demographics: age range 18-92 (mean 58.8, SD 11.79), 56% male, and 75% white. The median pedigree size was 21 (range 8-71) and the FHH entered into MeTree resulted in a database of 27,406 individuals. FHHs collected by MeTree were found to be high quality in 99.8% (N = 1,182/1,184) as compared to <4% at baseline. An average of 1.9 relatives per pedigree (range 0-50, SD 4.14) had no data reported. For pedigrees where at least one relative has no data (N = 497/1,184), 4.97 relatives per pedigree (range 1-50, SD 5.44) had no data. Talking with family members before using MeTree significantly decreased the proportion of relatives with no data reported (4.98% if you talked to your relative vs. 10.85% if you did not, p-value < 0.001.).

**Conclusion:**

Using MeTree improves the quantity and quality of the FHH data that is collected and talking with relatives prior to the collection of FHH significantly improves the quantity and quality of the data provided. This allows more patients to be accurately risk stratified and offered appropriate preventive care guided by their risk level.

**Trial number:**

NCT01372553

## Background

The systematic collection of family health history (FHH) can identify individuals at increased risk for common diseases [1-5]; many evidence-based guidelines rely upon risk assessment using FHH to guide the appropriate use of alternative (non-routine) screening procedures (such as breast MRI) and/or genetic counseling [6-9]. However, to use FHH for risk assessment, collected FHHs must have adequate detail. Unfortunately, studies have shown that the quality of FHH as currently collected in primary care is inadequate to assess disease risk [10-16]. Existing challenges include lack of patient preparation to provide FHH [17,18], the amount of time needed to collect FHH [19,20], lack of standardization, and limited training in synthesizing FHH data into a clinically actionable care plan [4,18,21,22]. The use of a FHH collection tool with risk assessment and clinical decision support (CDS) may address these challenges and increase the comprehensiveness of FHH collection and risk assessment in primary care [20].

To address these challenges the Genomedical Connection, a collaboration of Duke University, the University of North Carolina at Greensboro, and Cone Health, developed the Genomic Medicine Model with funding from the Department of Defense. The model [23,24], provides education to patients, providers, and community members; activates patients; and leverages a web-based software platform, MeTree [25], developed to promote accurate, high quality patient-entered FHHs for risk assessment in primary care. MeTree collects information about 48 diseases and provides risk assessment and CDS for five: breast, ovarian, and colon cancer, thrombophilia, and hereditary cancer syndromes. CDS documents include a 3 generation pedigree, a tabular FHH for PCPs, and two separate reports one for patients explaining their risk level and important points to discuss with their providers, and one for PCPs that indicates each individual’s level of risk, what put them at that risk, and what preventive actions can be taken to manage their risk [25]. This combination of patient education and integration with primary care is different from other FHH studies [26,27]. To assess the impact of the Genomic Medicine Model, MeTree was integrated into two primary care practices as part of a type II hybrid implementation-effectiveness controlled clinical trial [28,29]. This paper describes the content and quality of the FHHs collected using MeTree.

## Methods

### Patient recruitment

The protocol for the clinical trial has been previously published [23]. In brief, all adult patients scheduled for an upcoming well visit in two primary care practices were invited to participate. Only those who were adopted or did not speak English were excluded. Patients who agreed to participate were consented and provided with two brochures, one about how and why to collect FHH developed in conjunction with the Genetic Alliance, which included language about types of cancers and how to distinguish primary from secondary tumor sites, and another about disease risk and prevention. They were also given a worksheet with a list of relatives and a list and description of the conditions collected by MeTree to facilitate data collection from relatives prior to entering their information into MeTree. Further information about the development of these educational materials has been previously published [24]. A study coordinator was available to assist with questions. After patient s entered their FHH into MeTree, they completed a survey regarding who they talked to and what they learned when collecting their FHH. The study was approved by the IRBs of Duke University, University of North Carolina at Greensboro, Cone Health System, and the funding agency, the Department of Defense.

### FHH data and statistical analyses

FHH pedigree data entered into MeTree was stored in a SQL database and analyzed using R statistical software [30]. To define the characteristics and quality of the entered FHH we used the following criteria defined by Bennett [31]: (1) three generations of relatives, (2) relatives’ lineage (e.g. paternal or maternal side), (3) relatives’ gender, (4) an up-to-date FHH, (5) pertinent negatives in FHH noted (i.e. no FHH of cancer), (6) age of disease onset in affected relatives, and for deceased relatives, (7) the age and (8) cause of death. The nature of the study meant that all the FHHs were “up to date”; therefore, we limited analyses to the remaining 7 criteria. Hence in pedigrees with no deceased relatives we defined 5 possible criteria (excluding criteria 7 and 8 which related to deceased relatives) and in those with at least one deceased relative, we defined 7 possible criteria. For a pedigree to be considered high quality, at least one individual in the pedigree must meet all quality criteria (“high quality relative”), similar to previous study definitions [15]. To understand the impact of MeTree using broader definitions the percent of pedigrees meeting high quality criteria as a function of the number of “high quality” relatives is also reported.

Throughout this paper we present data in two ways:

1. **Data aggregated by proband** (enrolled patient): counts, proportions and averages for each proband’s pedigree were calculated and then further analyzed to show the distribution of counts, proportions and averages across all pedigrees. Data representing analysis of a pedigree will be annotated by the term “*pedigree*” throughout this paper and data describing the proportion of relatives meeting specific criteria will be termed “*proportion within pedigree*”.

2. **Data aggregated by all individuals** (enrolled patient and relatives): In this case, each person represents a data point, and all counts, proportions and averages represent the entire group of individuals without reference to which pedigree he or she belongs. As an example, for breast cancer prevalence the number of individuals listed as having breast cancer in the database was divided by the total number of individuals in the database. Data representing analysis of individuals without reference to the pedigree will be annotated by the term “*individual*” and data describing the proportion of individuals in the database meeting specific criteria will be termed “*prevalence across individuals*” without respect to pedigree membership.

To assess the generalizability of our population, we compared disease prevalence and heritability within our families to the general population using relative-type recurrence-risk ratios (lambdas) [32-34]. Lambdas represent the probability that a relative (e.g. sibling) will have a disease given the proband has the disease divided by the probability that the underlying population (in our case the enrolled patient) has the disease. We compare lambdas for our population to previously published population-based lambdas for siblings, since on average they share 50% of their DNA.

In calculation of quality criteria and disease prevalence, individuals were only counted once. For example, if a patient’s aunt had breast cancer and thrombosis, she would meet quality criterion for age of disease onset if the age of onset for only one of the diseases was entered. In addition, when calculating disease *prevalence across individuals* she would only be counted once for overall disease prevalence. But, when looking at individual diseases she would be counted in both the breast cancer and thrombosis categories. Numerical outcome variables were analyzed using multivariate analysis with standard linear regression, and categorical variables with logistic linear regression. Covariates evaluated include: age, gender, ethnicity, education level, family size, percent of family with cancer, proband’s perception of their knowledge of their FHH.

## Results

### Study enrollment

**Figure 1:**
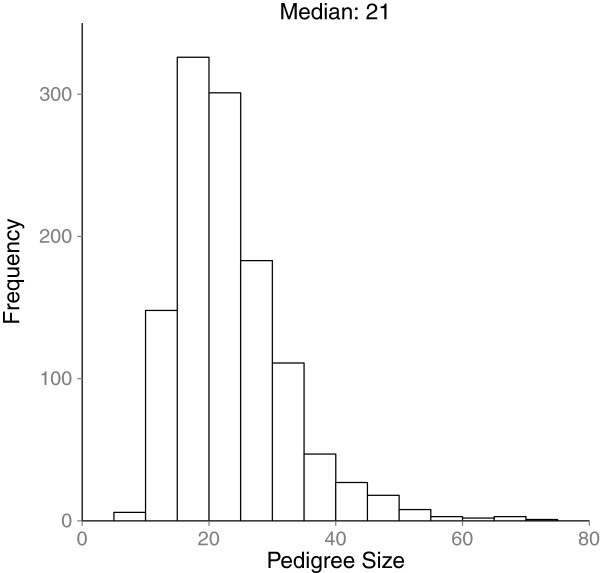
Mean pedigree size for each participant.

5,971 patients were contacted for participation in the study. 4,277 (72%) agreed to participate. However, given that there was only one designated computer in each clinic, only one participant per clinic could be enrolled per hour, thus 1,184 patients were enrolled and entered their FHH into MeTree. Enrollees were similar to the underlying clinic population with the exception of slightly more women and slightly fewer minorities than the general clinic population. (Table 1) Median pedigree size was 21 (range 8-71) (Figure 1) with 27,406 individuals entered in the database.

**Table 1:** Characteristics of patients enrolled to date as compared to the general clinic population*

	**Study patients (N = 1184) N (%)**	**Baseline clinic population (N = 45000) N (%)**
**Gender**		
Male	490 (41.4)	25,245 (56.1)
Female	694 (58.6)	19,215 (42.7)
**Ethnicity**		
White	969 (81.8)	33,840 (75.2)
Black	159 (13.5)	6,975 (15.5)
Other	56 (4.7)	4,230 (9.4)
**Age**		
Mean (SD)	58.8 (11.8)	59.3 (13.5)
<50	250 (21.1)	NA
50-65	575 (48.6)	NA
>65	359 (30.3)	NA
**Education**		
HS or less	158 (13.3)	NA
Some college	245 (20.7)	NA
College deg	461 (38.9)	NA
Any grad	320 (27.0)	NA

*previously published [28].

### Talking to relatives

53.89% (N = 638/1184) of patients contacted relatives to collect FHH. Those who contacted family talked to an average of 2.89 relatives (SD 1.58). Patients were more likely to contact family if they were women (57.35% vs. 48.98% of men, p = 0.004) or had more cancer in the family (15.15% vs. 12.15%, p < 0.001) and women (3.04 vs. 2.62 relatives for men, p < 0.001) and those with larger pedigrees (correlation = 0.13, slope = 0.02/relative, p < 0.001) talked to more relatives. For those who did not talk to their relatives, the most common reasons were: already knowing their FHH (N = 252/541, 46.58%), family not being available (N = 162/541, 29.94%), and having no time (N = 97/541, 17.92%).

### Perceptions of FHH knowledge

Regarding FHH knowledge the mean score was 4.30 (SD = 1.30) on a Likert scale (with 1 = no knowledge and 7 = knowing nearly everything). Knowledge perception was positively correlated with percent of pedigree with cancer (correlation = 0.16, slope = 1.97/percent increase, p-value < 0.001.) Women also tended to feel they had a higher knowledge of their FHH (4.46 vs. 4.08 for mean, p-value < 0.001).

### Quality criterion

MeTree ensures the first 4 FHH quality criteria are met: three generations, relatives’ lineage, relatives’ gender, and pertinent negatives. No predictors of quality were identified during multivariate analyses. Table 2 shows data comparing FHHs meeting each criteria with MeTree to that found during a chart review of FHH prior to study start [15]. In all categories except cause of death, MeTree FHHs met more criteria.

**Table 2:** Number (%) of Pedigrees that meet each quality criteria

	FHH* documentation prior to MeTree[15]	**MeTree**
	**N = 390 for all and 227 for deceased**	**N = 1184 for all and 1179 for deceased**
Quality criterion		
1. 3 generations of relatives	0 (0%)	1184 (100%)
2. Relatives’ lineage	111 (28.4%)	1184 (100%)
3. Relatives’ gender	356 (91.2%)	1184 (100%)
4. Pertinent negatives noted	173 (44.3%)	1184 (100%)
5. Age of disease onset	71 (18.2%)	854 (72.1%)
6. Cause of death	213 (98.1%)	695 (58.9%)
7. Age of death	172 (75.7%)	1156 (98.0%)

*FHH = family health history.

#### ***Age of disease onset***

The mean *proportion within pedigree* for reporting age of disease onset was 8% (range 0.0-40.0%, SD 7.0). Within the subgroup of pedigrees with at least one relative with age of disease onset reported (N = 854/1184, 72.13%) it was 10% (range 1.0-40.0%, SD 7.0).

#### ***Cause of death***

The mean *proportion within pedigree* for reporting the cause of death for deceased relatives was 12% (range 0.0-100%, SD 15.0). Within the subgroup of pedigrees with at least one relative with cause of death reported (N = 695/1179, 58.95%) it was 21% (range 3.0-100%, SD 15.0).

#### ***Age of death***

The mean *proportion within pedigree* for reporting the age of death on deceased relatives was 88% (range 0.0-100%, SD 23.0). Within the subgroup of pedigrees with at least one relative with age of death reported (N = 1156/1179, 98.05%) it was 89% (range 9.0-100%, SD 19.0).

#### ***Age and cause of death***

The mean *proportion within pedigree* for reporting both the age and cause of death on deceased relatives was 6% (range 0.0-100%, SD 11.0). Within the subgroup of pedigrees with at least one relative with age and cause of death reported (N = 479/1179, 40.62%) it was 16% (range 3.0-100%, SD 11.0).

### High quality pedigrees

**Figure 2:**
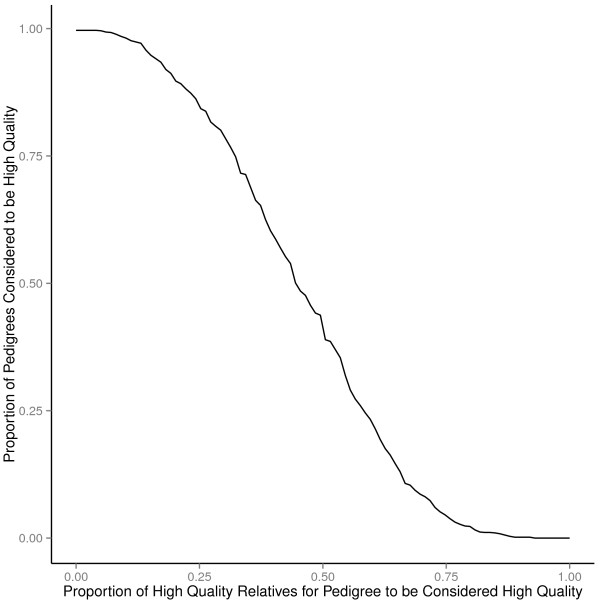
Percent of high-quality pedigrees as percent of relatives with high-quality pedigrees required increases.

FHHs collected by MeTree were high quality in 99.83% (N = 1182/1184) when requiring only one relative to meet all the quality criteria. Figure 2 shows how the proportion of high quality FHHs changes as the proportion of relatives required to meet all of the quality criteria (high quality relatives) increases from 0 to 1. Even when 40% of the pedigree must contain high quality relatives, over 60% of the FHHS were still high quality.

### Relatives with no reported data

Given how MeTree structures FHH collection, it is not possible to differentiate relatives who are healthy, and therefore do not have any diseases, from relatives for whom the patient does not know their medical history. Since we cannot distinguish, we report the frequency of having no data reported for any given relative (*“no data”*) and for relative categorized by degree of relatedness to the patient.

For all pedigrees the *proportion within pedigree* of *no data* relatives was 8% (range 0.0-8.6%, SD 1.4) (an average of 1.9 relatives/pedigree (range 0-50, SD = 4.14)). For the subgroup of pedigrees with at least one *no data* relative (N = 452/1184) the proportion increased to 20% (range 1.0-50.0%, SD = 5.4) or 4.97 relatives/pedigree (range 1-50, SD = 5.44). Having a lower *proportion within pedigree of no data* relatives was associated with FHH knowledge (correlation = -0.19, slope = -0.02/point increase in FHH knowledge, p-value < 0.001) and talking with family members (4.98% if talked to a relative vs. 10.85% if did not, p-value < 0.001). Talking with family members also increased the number of pedigrees for which 100% of relatives had at least some information entered (70.38% if talked to a relative vs. 51.83% if did not, p-value < 0.001). Multivariate analyses indicated that African-Americans (OR = 3.24, CI 1.95-5.41, p-value < 0.001) and older patients (OR = 1.04, CI 1.01-1.06, p-value < 0.001) were more likely to have relatives with *no data*; but having a larger pedigree had no effect.

#### ***First degree relatives (FDR)***

On average the *proportion within pedigree* of FDRs with *no data* (e.g. parents, siblings, children) was 1% (range 0.0-89%, SD 5.0). However, only 30 pedigrees of the total 1,184 had an FDR with *no data*, and among this small subgroup the *proportion* was much higher at 26% (range 7.0-89%, SD 17.0).

#### ***Second degree relatives (SDR)***

A larger percentage of SDRs (e.g. grandparents, aunts) had *no data*, 11% (range 0.0-100%, SD = 21.0). Among pedigrees with at least one SDR with *no data* (N = 447/1184) the mean proportion was 30% (range 2.0-100%, SD = 25.0). Grandparents were the most likely SDR to have *no data* (mean = 16%, range 0.0-1.00, SD = 28.0) and among pedigrees with at least one grandparent with *no data* (N = 345/1184) the proportion increased to 54% (range 25.0-100%, SD = 25.0).

### Deceased relatives

The mean *proportion within pedigree* of deceased relatives was 46% (range 0.0-95%, SD = 18.0); only 5 pedigrees had no deceased relatives. *Prevalence across individuals* for deceased relatives was 44.75% (N = 12,264). Of those who were deceased, 30% were FDRs and 59% SDRs (of which 42% were grandparents).

### Disease proportion and risk recurrence ratios

When looking at *probands* only, the disease prevalence rates were: breast cancer 6.48% (N = 45/694), colon cancer 0.42% (N = 5/1184), diabetes 9.29% (N = 110/1184), heart attack 3.04% (N = 36/1184). This is comparable to age adjusted national prevalence rates for: diabetes (9.4%) [35], heart disease (6.4%) (which includes heart attack as well as other forms of heart disease) [36], and colon cancer (0.50%) [37]; though the rate of breast cancer was higher than the national rate (3.7%) [37]. When looking at *prevalence across individuals* the rates were as follows: ovarian cancer 0.97% (N = 133/13,659 females), breast cancer 4.71% (N = 644/13,659 females) with an additional 2 men, colon cancer 1.37% (N = 376/27,406), hereditary cancer syndromes 0.91% (N = 250/27,406), thrombosis 1.76% (N = 482/27,406), “heart attacks” 8.76% (N = 2400/27,406), diabetes 6.36% (N = 1744/27,406), asthma 2.82% (N = 744/27,406), and dementia 3.60% (N = 987/27,406). Sibling recurrence-risk ratios were as follows: breast cancer 2.61 (CI 1.29-4.65), colon cancer 27.86 (CI 0-91.22), heart attack 5.89 (CI 2.44-10.49), and diabetes 2.49 (CI 1.79-3.18), which are similar to reported population values for: breast cancer (1.8), colon cancer (2.7), ischemic heart disease (2.0), and diabetes (5.3) [38].

## Discussion

MeTree provides a significant improvement in the quality and content of FHH collection and thereby improves our ability to perform risk assessment and appropriately identify individuals meeting criteria for “non-routine” screening or diagnostic strategies based on current clinical guidelines. Other structured tools such as Health Heritage have also found improvements in collection of FHH and the ability to provide risk assessments [26,39,40]. We found that by educating patients about how and what to collect for FHH, patients talked with relatives and were able to give more, higher quality information. Those who talked with their relatives and those with higher perceptions of their FHH knowledge were more likely to have data reported on all family members and have a lower proportion of relatives with no data entered (*no data* relatives). As the amount and the quality of data on each relative improves, the ability to perform accurate risk assessment is also improved; and, as shown by the fact that over 60% of MeTree pedigrees had at least 40% of relatives meeting all the quality criteria (Figure 2), it will allow risk assessments to occur at higher rates than what currently occurs in practice.

To assess the generalizability of our findings, we compared the data to published population values. In particular, we wanted to evaluate whether those who participated were more likely to have a disease or have family members with a disease that would activate them, making them more likely to report a higher quality FHH than the general population. We found that those who participated in this study had similar disease prevalence for colon cancer [37] and diabetes [35] but a slightly higher prevalence of breast cancer [37] as compared to national statistics. They also had a higher disease prevalence *within their families* for colon cancer [41] and breast cancer [41,42]. However, sibling recurrence-risk ratios, which take into consideration the relationships of affected members within families, were consistent with reported population values across all diseases [38]. From this we conclude that because the probands were demographically similar to the underlying clinic population, to the disease prevalence in the general U.S. population, and to the sibling risk-ratios in the general U.S. population, our findings are likely to be generalizable despite being slightly enriched with probands with breast cancer. In addition, because few probands had any of the diseases (even though the percentage for breast cancer was higher), the effect of a recruitment bias, if present, is expected to be minimal.

There are some other biases that must be taken into account in the interpretation of this data. A higher percentage of patients’ FHHs collected by primary care providers (PCP) [15] (as seen in the baseline chart review) contained cause of death information than those collected in MeTree. It is highly probable that this is a result of reporting bias. In the baseline chart review, 71.8% (N = 163/227) of pedigrees had no deceased relatives mentioned at all; while with MeTree < 1% (N = 5/1184) had no deceased relatives. This highlights the difference in the type of information collected by providers. When providers do report a deceased relative they almost always report cause of death; however, they only rarely capture deceased relatives. The source of this bias is likely that providers tend to be disease focused. If patients do not know what a family member died of, providers are unlikely to make note of the death at all. In contrast the entire family structure is known with MeTree, as are all the relatives who are deceased, even if the cause of death is not known. Currently, no risk assessment algorithms take in to consideration a relative’s cause of death; only their age of death, age of disease onset, and presence of disease are considered. While this may change in the future and the collection of this information needs to be improved, it does not, at the moment, affect ability to assess risk.

Incomplete second degree relative (SDR) data poses another limitation, as SDR data, and grandparents in particular, are used in risk assessment calculations, particularly for colon cancer screening and hereditary cancer syndrome risk [43]. The Family Healthware Intervention Trial (FHITr) also found that reporting of “don’t know” responses was significantly higher for second- versus first-degree relatives (FDR) for all six diseases assessed [40] at rates consistent with what we found (1% for FDR vs. 11% for SDR). We also found that probands who did not know the health history of one grandparent were much more likely not to know the others. This was improved significantly among those who talked with their relatives (10% vs. 23%, p-value < 0.001) and suggests that reporting can be improved by encouraging family discussions. However, this problem is likely to persist because of ongoing generational biases against discussing medical problems and the increasing separation of family units in today’s society.

## Conclusion

In conclusion, MeTree provides PCPs with a higher quality FHH than they are able to collect on their own, by compensating for the lack of patient preparation and the time constraints of the clinic visit. The amount of data entered and the quality of the data were sufficient to perform risk assessments on the vast majority of patients, allowing providers to focus on review of FHH (instead of collection) and risk assessment and intervention plans based on that knowledge—both from their own knowledge of risk and on the guideline-based recommendations from MeTree.

## Competing interests

All authors declare that they have no competing interest to report.

## Authors’ contributions

RW participated in interpretation of data, drafted and critically revised the manuscript, and gave final approval of the current version. TH analyzed and assisted in interpretation of the data, assisted in drafting the manuscript, and gave final approval of the current version. AB contributed to concept and design of the study, critically revised the manuscript, and gave final approval of the current version. KP assisted in data acquisition and interpretation, critically revised the manuscript, and gave final approval of the current version. EH contributed to analysis and interpretation of the data, critically revised the manuscript, and gave final approval of the current version. GG contributed to conception and design of the study, critically revised the manuscript, and gave final approval of the current version. VH contributed to conception and design of the study, critically revised the manuscript, and gave final approval of the current version. LO contributed to study design, interpretation of data, critically revised the manuscript, and gave final approval of the current version. RW and LO had full access to all the data in the study and takes responsibility for the integrity of the data and the accuracy of the analysis. All authors have no conflicts of interest to report.

## Pre-publication history

The pre-publication history for this paper can be accessed here:

http://www.biomedcentral.com/1471-2296/15/31/prepub
